# Recommendations for HIV Screening of Gay, Bisexual, and Other Men Who Have Sex with Men — United States, 2017

**DOI:** 10.15585/mmwr.mm6631a3

**Published:** 2017-08-11

**Authors:** Elizabeth A. DiNenno, Joseph Prejean, Kathleen Irwin, Kevin P. Delaney, Kristina Bowles, Tricia Martin, Amrita Tailor, Gema Dumitru, Mary M. Mullins, Angela B. Hutchinson, Amy Lansky

**Affiliations:** 1 Division of HIV/AIDS Prevention, National Center for HIV/AIDS, Viral Hepatitis, STD, and TB Prevention, CDC.

CDC’s 2006 recommendations for human immunodeficiency virus (HIV) testing state that all persons aged 13–64 years should be screened for HIV at least once, and that persons at higher risk for HIV infection, including sexually active gay, bisexual, and other men who have sex with men (MSM), should be rescreened at least annually (*1*). Authors of reports published since 2006, including CDC (*2*), suggested that MSM, a group that is at highest risk for HIV infection, might benefit from being screened more frequently than once each year. In 2013, the U.S. Preventive Services Task Force (USPSTF) found insufficient evidence to specify an HIV rescreening interval but recommended annual screening for MSM as a reasonable approach (*3*). However, some HIV providers have begun to offer more frequent screening, such as once every 3 or 6 months, to some MSM. A CDC work group conducted a systematic literature review and held four expert consultations to review programmatic experience to determine whether there was sufficient evidence to change the 2006 CDC recommendation (i.e., at least annual HIV screening of MSM in clinical settings). The CDC work group concluded that the evidence remains insufficient to recommend screening more frequently than at least once each year. CDC continues to recommend that clinicians screen asymptomatic sexually active MSM at least annually. Each clinician can consider the benefits of offering more frequent screening (e.g., once every 3 or 6 months) to individual MSM at increased risk for acquiring HIV infection, weighing their patients’ individual risk factors, local HIV epidemiology, and local testing policies.

HIV testing is the critical first step in making HIV-infected persons aware of their status, so that they can obtain treatment and prevent transmission of HIV. In 2014, CDC estimated that 15% of all persons living with HIV in the United States had undiagnosed infections (*4*). Early HIV care and adherence to antiretroviral therapy (ART) prolong life and decrease the chances of HIV transmission (*5*). The increasing availability of antigen-antibody HIV tests means that a greater number of infections can be detected in the highly infectious, acute stage of infection (*6*). The potential benefits of early detection and treatment of HIV were the driving force behind CDC’s initiative to assess the benefits and harms associated with more frequent screening of MSM. This policy note describes the results of that initiative.

## Systematic Review

A CDC work group of federal employees comprising a diverse group of epidemiologists, clinicians, behavioral scientists, health policy experts, and health economists was convened. To identify studies comparing annual versus more frequent screening among MSM, the CDC work group conducted a systematic literature review, using methods adapted from the Guide for Community Preventive Services (*7*,*8*), and convened four consultations with 24 external experts to obtain their individual input on the programmatic and scientific evidence. During 2013–2014, and updated in January 2015, the CDC work group conducted a systematic review of published studies indexed in MEDLINE, EMBASE, PsycINFO, and CINAHL. The search was restricted to articles that 1) were published during 2005–2014 (last search conducted in January 2015); 2) described analyses conducted in the United States, Canada, Australia, New Zealand, and Western Europe; and 3) contained the following search terms: HIV seropositivity, HIV infection, AIDS serodiagnosis, sexually transmitted diseases/infections, men who have sex with men (MSM), high risk, test, screen. Included articles provided information on one of four outcomes of interest: 1) health benefits to individual MSM being screened or to the community (e.g., averted secondary HIV infections); 2) harms to individual MSM (stigma or out-of-pocket costs); 3) acceptability (MSM attitudes toward more frequent screening); or 4) feasibility (barriers to or facilitators of state or local screening). Included studies were restricted to those conducted in clinical settings. A manual search of gray literature was also conducted.

The CDC work group reviewed 6,479 abstracts resulting from the automated search, 111 of which met the initial screening inclusion criteria and were reviewed in full. Three members of the CDC work group, working in overlapping pairs, applied inclusion criteria to these studies, rating each study for outcome (benefits, harms, acceptability, or feasibility). They used a quantitative study assessment tool to note key findings. Discrepancies were resolved by a third reviewer who was not a member of the original pair (*7*,*8*).

Thirteen studies met the inclusion criteria and were evaluated on quality of evidence (*9*). For each of the four study outcomes, CDC HIV testing experts then evaluated the quality of evidence to determine design suitability (high, moderate, or low), execution (good, fair, or poor), and consistency of study results, with one exception: the eight mathematical modeling studies were not rated on quality of execution because of the lack of a grading system appropriate for the different mathematical model types included.

Overall, the quality of studies was low. Eleven studies addressed health or economic benefits of more frequent screening compared with annual screening. Eight of these were mathematical models that the CDC work group classified as having low suitability because of uncertainty about the validity of the parameter estimates and questions about the models’ generalizability. Two studies addressed intervals between HIV screening or diagnostic tests in clinical settings, but did not directly address the acceptability of more frequent than annual HIV screening among asymptomatic MSM. No studies addressed harms associated with, or the feasibility of, conducting more frequent HIV screening in clinical settings in the United States. Additional details about these studies can be found elsewhere (*9*).

After deliberations that involved discussion, consensus building, and voting, the CDC work group concluded that insufficient evidence exists in the published and unpublished literature to warrant changing CDC’s current recommendation to offer HIV screening at least annually to all sexually active MSM.

## Expert Consultation Series Results

During August–December 2014, the CDC work group convened a series of consultations with external subject matter experts, including clinicians, epidemiologists, academic researchers, health department policy and program staff members, and members of the MSM community, to 1) obtain their individual input on the results of the systematic review and preliminary conclusions; 2) obtain the opinions and experiences of experts from three public-sector HIV screening programs that provided more frequent than annual HIV screening to MSM; and 3) identify studies missed in the literature review or data that could be analyzed in the future to inform recommendations about HIV screening frequency.

Postconsultation analysis of the individual feedback from experts revealed that most believed the literature was insufficient to conclude that more frequent screening had demonstrated benefits over annual screening but that the scientific and programmatic evidence suggested that some MSM would be willing to be screened more frequently. Experts from health departments already implementing more frequent than annual screening described benefits of their programs, including decreases in the proportion of MSM with undiagnosed HIV infection. Experts also individually agreed that the estimates from the mathematical models suggest a benefit to more frequent screening, particularly in jurisdictions providing prompt, high-quality access to HIV medical care, where early treatment with ART decreases infectiousness and would likely decrease the number of new HIV infections in sex or drug-using partners. In addition, individual experts stressed the importance of the cost-effectiveness modeling studies, which estimated that more frequent screening, compared with annual screening, would be more cost-effective by averting new HIV infections (incremental cost-effectiveness ratio, range = cost-saving – $138,200/quality-adjusted life year) (*9*). Finally, most experts stated that mathematical models do not provide sufficient evidence to warrant by themselves a change in the guideline, because of limitations in their study design, and that additional studies are needed to update the current recommendation.

## Recommendations

CDC concludes that the evidence, programmatic experience, and expert opinions are insufficient to warrant changing the current recommendation (annual screening for MSM) to more frequent screening (every 3 or 6 months). Therefore, CDC’s 2006 recommendation for HIV screening of MSM is unchanged; providers in clinical settings should offer HIV screening at least annually to all sexually active MSM. Clinicians can also consider the potential benefits of more frequent HIV screening (e.g., every 3 or 6 months) for some asymptomatic sexually active MSM based on their individual risk factors, local HIV epidemiology, and local policies (*9*). Additional research is needed to establish the individual- or community-level factors that might increase the risk for HIV acquisition for MSM and merit more frequent HIV screening. For MSM who are prescribed preexposure prophylaxis, HIV testing every 3 months and immediate testing whenever signs and symptoms of acute HIV infection are reported (*10*) is indicated. MSM who experience a specific high-risk sexual exposure or have symptoms of recent HIV infection should seek immediate HIV testing, and clinicians should be alert for the symptoms of acute HIV infection and provide appropriate diagnostic testing.

CDC encourages researchers to conduct studies to evaluate the benefits and harms of more frequent screening for MSM. Findings from these studies will inform future assessment of recommendations. CDC will continue to monitor the evidence on the effectiveness of various HIV screening intervals and consider the need to revise current recommendations in light of new evidence.

## References

[1] Branson BM, Handsfield HH, Lampe MA, Revised recommendations for HIV testing of adults, adolescents, and pregnant women in health-care settings. MMWR Recomm Rep 2006;55(No. RR14). 16988643

[2] Oster AM, Miles IW, Le BC, HIV testing among men who have sex with men—21 cities, United States, 2008. MMWR Morb Mortal Wkly Rep 2011;60:694–9. 21637183

[3] Moyer VA; U.S. Preventive Services Task Force. Screening for HIV: U.S. Preventive Services Task Force recommendation statement. Ann Intern Med 2013;159:51–60. 10.7326/0003-4819-159-1-201307020-00645 23698354

[4] CDC. HIV Monitoring selected national HIV prevention and care objectives by using HIV surveillance data—United States and 6 dependent areas, 2015. HIV surveillance supplemental report vol. 22, no. 2. Atlanta, GA: US Department of Health and Human Services, CDC, National Center for HIV/AIDS, Viral Hepatitis, STD, and TB, Division of HIV/AIDS Prevention; 2017. https://www.cdc.gov/hiv/pdf/library/reports/surveillance/cdc-hiv-surveillance-supplemental-report-vol-22-2.pdf

[5] INSIGHT START Study Group. Initiation of antiretroviral therapy in early asymptomatic HIV infection. N Engl J Med 2015;373:795–807. 10.1056/NEJMoa1506816 26192873 PMC4569751

[6] CDC; Association of Public Health Laboratories. Laboratory testing for the diagnosis of HIV infection: updated recommendations. Atlanta, GA: US Department of Health and Human Services, CDC; 2014. https://stacks.cdc.gov/view/cdc/23447

[7] Briss PA, Zaza S, Pappaioanou M, ; Task Force on Community Preventive Services. Developing an evidence-based Guide to Community Preventive Services—methods. Am J Prev Med 2000;18(Suppl):35–43. 10.1016/S0749-3797(99)00119-1 10806978

[8] Zaza S, Wright-De Agüero LK, Briss PA, ; Task Force on Community Preventive Services. Data collection instrument and procedure for systematic reviews in the Guide to Community Preventive Services. Am J Prev Med 2000;18(Suppl):44–74. 10.1016/S0749-3797(99)00122-1 10806979

[9] DiNenno EA, Prejean J, Delaney K, Evaluating the evidence for more frequent than annual HIV screening of gay, bisexual, and other men who have sex with men in the United States: results from a systematic review and CDC expert consultation. Public Health Rep 2017. In press. 10.1177/0033354917738769 PMC5805092 29182894

[10] US Public Health Service. Preexposure prophylaxis for the prevention of HIV infection in the United States—2014: a clinical practice guideline. Washington, DC: US Department of Health and Human Services, US Public Health Service; 2014. https://www.cdc.gov/hiv/pdf/PrEPguidelines2014.pdf

